# Early chains of transmission of COVID-19 in France, January to March 2020

**DOI:** 10.2807/1560-7917.ES.2022.27.6.2001953

**Published:** 2022-02-10

**Authors:** Juliette Paireau, Alexandra Mailles, Catherine Eisenhauer, Franck de Laval, François Delon, Paolo Bosetti, Henrik Salje, Valérie Pontiès, Simon Cauchemez

**Affiliations:** 1 Mathematical Modelling of Infectious Diseases Unit, Institut Pasteur, Université de Paris, CNRS UMR2000, Paris, France; 2 Santé publique France, French National Public Health Agency, Saint-Maurice, France; 3 SSA, Service de Santé des Armées, CESPA, French Armed Forces Center for Epidemiology and Public Health, Marseille, France; 4 Aix Marseille Univ, INSERM, IRD, SESSTIM, Sciences Economiques & Sociales de la Santé & Traitement de l’Information Médicale, ISSPAM, Marseille, France; 5 Department of Genetics, University of Cambridge, Cambridge, United Kingdom; 6 Santé publique France, Hauts-de-France Regional Office, Lille, France

**Keywords:** SARS-CoV-2, transmission, contact tracing, cluster, secondary clinical attack rate, superspreading

## Abstract

**Introduction:**

SARS-CoV-2, the virus that causes COVID-19, has spread rapidly worldwide. In January 2020, a surveillance system was implemented in France for early detection of cases and their contacts to help limit secondary transmissions.

**Aim:**

To use contact-tracing data collected during the initial phase of the COVID-19 pandemic to better characterise SARS-CoV-2 transmission.

**Methods:**

We analysed data collected during contact tracing and retrospective epidemiological investigations in France from 24 January to 30 March 2020. We assessed the secondary clinical attack rate and characterised the risk of a contact becoming a case. We described chains of transmission and estimated key parameters of spread.

**Results:**

During the study period, 6,082 contacts of 735 confirmed cases were traced. The overall secondary clinical attack rate was 4.1% (95% confidence interval (CI): 3.6–4.6), increasing with age of index case and contact. Compared with co-workers/friends, family contacts were at higher risk of becoming cases (adjusted odds ratio (AOR): 2.1, 95% CI: 1.4–3.0) and nosocomial contacts were at lower risk (AOR: 0.3, 95% CI: 0.1–0.7). Of 328 infector/infectee pairs, 49% were family members. The distribution of secondary cases was highly over-dispersed: 80% of secondary cases were caused by 10% of cases. The mean serial interval was 5.1 days (interquartile range (IQR): 2–8 days) in contact tracing pairs, where late transmission events may be censored, and 6.8 (3–8) days in pairs investigated retrospectively.

**Conclusion:**

This study increases knowledge of SARS-CoV-2 transmission, including the importance of superspreading events during the onset of the pandemic.

## Introduction

Coronavirus disease 2019 (COVID-19) emerged in Wuhan, China in December 2019 and has since spread globally. The disease is caused by severe acute respiratory syndrome coronavirus 2 (SARS-CoV-2) and is transmitted from person to person mainly via small droplets produced by coughing, sneezing or talking. The rapid spread of the virus across the world has led to unprecedented containment measures, with 4.5 billion people being confined at home [1]. Following a fast rise in intensive care unit admissions, France went into lockdown on 17 March 2020 for 7 weeks. As of 13 May 2021, 5,821,668 cases have been confirmed, which includes 459,339 hospitalisations and 106,964 deaths [2].

In the early phase of the epidemic, France attempted to contain imported SARS-CoV-2 infections. On 10 January 2020, a dedicated surveillance system was implemented to allow early detection of cases and their contacts, limit secondary transmission and slow the spread of the virus. Upon detection of a COVID-19 case, contact tracing was initiated and a follow-up procedure was implemented. The first three COVID-19 cases were detected on 24 January 2020 in travellers returning from Wuhan [3].

Contact tracing is an essential tool to control epidemics and has proven effective in the past [4,5]. Contact tracing can also improve our knowledge on transmission dynamics and the natural history of emerging pathogens such as SARS-CoV-2. Contact tracing studies can provide estimates of secondary attack rates, explore risk factors of infection among contacts and assess the relative contributions of different types of contact to transmission, information that can be used to define effective control strategies [6-12]. In addition, analysing chains of transmission can be used to identify who infected whom, to document the characteristics of infectors and infectees and the types of contact between them [6], and to assess the contribution of super-spreading events [13], information that population-level data cannot provide. Analysing chains of transmission is particularly useful for estimating key parameters of spread, which is essential information for mathematical models. Moreover, it can be useful to compare these estimates across diverse locations, time periods and settings.

Here, we analysed the detailed data collected during contact tracing and retrospective epidemiological investigations in the early phase of the pandemic in France, from 24 January 2020 to 30 March 2020 (a national lockdown was implemented on 17 March 2020). Our study had four objectives: to assess the secondary clinical attack rate; to identify the factors associated with the risk of a contact becoming a case; to describe chains of transmission; and to estimate key parameters of spread.

## Methods

### Surveillance system

Implemented on 10 January 2020, the individual-based surveillance system for COVID-19 was designed to detect cases as early as possible (see [3] for a detailed description). Possible cases were isolated in one of the COVID-19 reference hospitals when possible or at home. These cases were interviewed using a standardised questionnaire that collected information on sociodemographic characteristics, clinical characteristics and history of exposure (including potential infectors). Data were entered into a secure web-based application (Go.Data, World Health Organization). Respiratory samples were taken from all possible cases and tested for SARS-CoV-2 using reverse transcription polymerase chain reaction (RT-PCR).

From 17 to 29 January 2020, a possible case was defined either as a patient with a severe acute lower respiratory infection requiring admission to hospital and with a history of travel to or residence in Wuhan, China in the 14 days before symptom onset or a patient with an acute respiratory illness irrespective of severity and with a history of at-risk exposure, mainly with a confirmed case [3]. A confirmed case was defined as a possible case with a positive RT-PCR. Possible cases who tested negative were classified as excluded cases. This case definition slightly evolved during the study period to adapt to the epidemiological situation. The detailed case definition used at the start of the pandemic as well as the case definition in effect at the end of the study period are listed in the Supplementary Tables S1 and S2.

From 14 March 2020, the system was replaced by a population-level approach, which was more adapted to the growing size of the epidemic. However, individual-based surveillance continued for a few more days in less affected regions.

### Contact tracing

Confirmed cases were kept in isolation and interviewed about any contacts that occurred during the time they were symptomatic and the day before symptom onset, in accordance with European recommendations [14]. Based on their type of exposure, identified contacts were classified into three levels of exposure risk: negligible, low or moderate/high risk (Table). Only contacts who developed symptoms compatible with COVID-19 were tested for SARS-CoV-2 using RT-PCR. If the test was positive, they were considered secondary confirmed cases and their contacts were then traced in the same fashion as a primary case. Following the procedures, only contacts with low or moderate/high risk were followed up (Table). However, because the investigation teams were quickly overloaded in the exponential growth phase of the epidemic, most contacts (97%) who were identified and entered into the database were moderate/high-risk contacts. In regions heavily affected by the epidemic, some cases had their contacts traced but not entered into the web-based application. That is, the database is not exhaustive as it represents only a sample of all the contact tracing efforts. The number of contacts not entered in the database is unknown. In some of these regions, contact tracing became too difficult to conduct and was stopped before 14 March 2020.

**Table:** Definition of contacts of COVID-19 cases and follow-up procedures by level of exposure risk, France, January 2020

Level of exposure risk	Contact definition	Follow-up procedure
Negligible risk	Person who had short (< 15 min) contact with a confirmed case in public settings such as in public transportation, restaurants and shops; healthcare personnel who treated a confirmed case while wearing appropriate PPE without any breach identified.	Neither identification nor information of contacts.
Low risk	Person who had a close (within 1 m) but short (< 15 min) contact with a confirmed case, or a distant (> 1 m) but prolonged contact in public settings, or any contact in private settings that does not match with the moderate/high risk of exposure criteria.	Contacts are asked to measure their body temperature twice a day and check for clinical symptoms. If contacts have symptoms such as fever, cough, or dyspnoea, they are asked to wear a surgical mask, self-isolate, and immediately contact the emergency hotline (SAMU-centre 15) and identify themselves as contacts of a confirmed COVID-19 case.
Moderate/ high risk	Person who had prolonged (> 15 min) direct face-to-face contact within 1 m with a confirmed case, shared the same hospital room, lived in the same household or shared any leisure or professional activity in close proximity with a confirmed case, or travelled together with a COVID-19 case in any conveyance without appropriate individual protection equipment. Healthcare personnel who treated a confirmed case without wearing appropriate PPE or with an identified breach.	In addition to the above, contacts are asked to stay at home for 14 days after their last contact with the confirmed case while symptomatic and to avoid contacts with the other persons living in the same household (or at least wear a surgical mask). The follow-up consists of an active follow-up through daily calls from the regional follow-up team organised by the Regional Health Agency in collaboration with Santé publique France.

COVID-19: coronavirus disease; PPE: personal protective equipment.

Adapted from [3].

### Retrospective epidemiological investigations

In addition to the chains of transmission established through prospective contact tracing, some infector/infectee pairs were reconstituted retrospectively during epidemiological investigations. This was especially the case in the Oise department in northern France, where a large cluster of COVID-19 cases was detected in February 2020 [15]. As a result, a thorough investigation was conducted in this department to reconstruct the chains of transmission responsible for the cluster and to understand the rapid spread of the pathogen (Supplementary Text S1).

### Statistical analysis

We first analysed contact tracing data for confirmed cases (hereafter called cases) and their contacts, who were entered into the database between 24 January 2020 and 30 March 2020. We defined an index case as a case whose detection initiated contact tracing. Therefore, secondary cases can become index cases if their contacts are traced.

We described contact patterns between index cases and their traced contacts by constructing the corresponding contact matrix. We compared this to the age-specific contact matrix for the French population obtained from the COMES-F study performed in 2012 [16]. We analysed the differences in the age-specific mixing patterns using linear regression.

We estimated the secondary clinical attack rate – i.e., the proportion of symptomatic cases among the contacts of an index case [17]. We investigated the factors associated with the risk of a contact becoming a case (i.e., developing symptoms and testing positive) using multivariable logistic regression (Supplementary Text S2).

Finally, we analysed all infector/infectee pairs using pairs identified through prospective contact tracing (pairs between an index case and a contact who became a case) and pairs identified through retrospective epidemiological investigations in Oise. We computed the mean number of secondary cases generated by each case based on contact tracing data (using the number of secondary cases observed among traced contacts of each index case) or retrospective data (where cases at the end of the observed chain of transmission were considered to have no secondary cases) (Supplementary Text S3). We also estimated the serial interval – i.e., the interval between symptom onset in infector and infectee in days (Supplementary Text S3).

Distributions were compared using Wilcoxon-Mann-Whitney test and proportions were compared using chi-squared test. Results with p-value < 0.05 were considered significant. Percentages were based on those for whom information was available. All analyses were performed in R software [18].

### Ethical statement

The investigations were carried out in accordance with the General Data Protection Regulation (Regulation (EU) 2016/679 and Directive 95/46/EC) and French data protection law (Law 78–17 on 06/01/1978 and Décret 2019–536 on 29/05/2019). No specific ethical approval was needed as this investigation was covered by the authorization delivered to Santé publique France by the French data protection authority (CNIL) to process personal health data in order to prevent, alert or monitor an epidemiological crisis (authorization 341194 V42).

## Results

### Description of contact tracing data

Between 24 January and 30 March 2020, 6,082 contacts (6,028 unique individuals) of 735 cases were traced and entered into the database. The median age of the index cases was 50 years (interquartile range (IQR): 36–65) and 52% (384/734) were female. The median age of the contacts was 38 years (IQR: 21–55) and 56% (3,123/5,593) were female.

**Figure 1:**
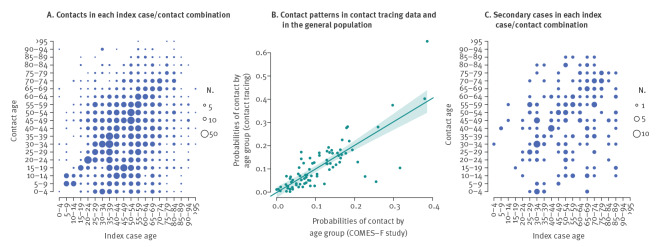
Distribution of COVID-19 cases (n = 735) and contacts (n = 6,028) by age group, France, 24 January 2020–30 March 2020

Cases had 8.3 contacts traced on average (median 4, range 1–146). We observed non-random contact patterns by age: the index cases tended to have more traced contacts of similar age (Pearson correlation coefficient (r) = 0.32, p < 0.01) (Figure 1A). These patterns were consistent with age-specific contacts measured before the COVID-19 pandemic in the French general population [16] (r = 0.80, p < 0.01) (Figure 1B and Supplementary Figure S1). The infector/infectee pairs followed a similar assortative pattern by age (r = 0.29, p < 0.01) (Figure 1C).

COVID-19: coronavirus disease.

Panel B depicts the correlation between contact patterns in the contact tracing data and contact patterns in the French general population from the COMES-F study [16]: each point represents one entry of the contact matrices (represented in Supplementary Figure S1) and corresponds to the probability for a given age group to have a contact with another age group; contact matrices were compared using 10-year age groups.

**Figure 2:**
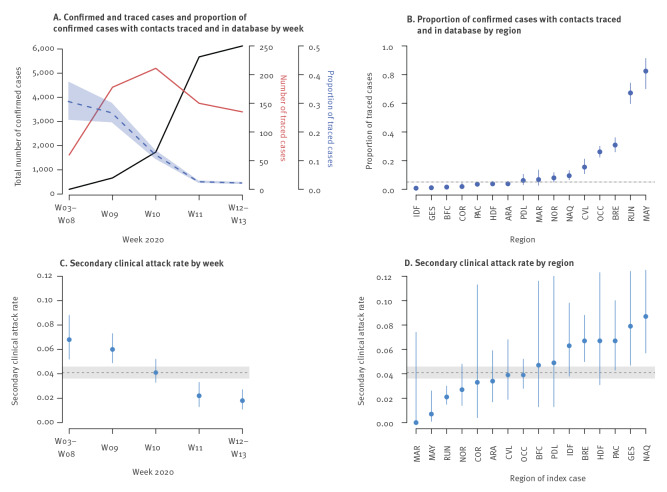
Description of contact tracing data for COVID-19 cases over time and regions, France, 24 January 2020–30 March 2020 (n = 735 index cases; 6,028 contacts; 248 secondary cases)

COVID-19: coronavirus disease; W: week.

ARA: Auvergne-Rhône-Alpes; BFC: Bourgogne-Franche-Comté; BRE: Bretagne; CVL: Centre-Val de Loire; COR: Corse; GES: Grand Est; HDF: Hauts-de-France; IDF: Île-de-France; MAR: Martinique; MAY: Mayotte; NAQ: Nouvelle-Aquitaine; NOR: Normandie; OCC: Occitanie; PAC: Provence-Alpes-Côte d’Azur; PDL: Pays de la Loire; RUN: La Réunion

The 735 cases who had their contacts traced and entered into the database represented 5% (735/14,400) of the total number of COVID-19 cases over the study period. This proportion decreased over time, from 31% at the beginning of the epidemic to 2% in weeks 12–13 (week 11 was the last week of the individual based-surveillance at the national level) (Figure 2A). The proportion of cases who had their contacts traced and entered into the database also varied by region (Figure 2B). The most heavily affected regions (Ile-de-France and Grand Est), representing 54% of cases, had the lowest proportion of traced cases (1%).

Week 11 was the last week of the individual-based surveillance at the national level. The horizontal dashed lines indicate the national values over the whole time period. Shaded areas and vertical bars represent 95% confidence intervals.

### Secondary clinical attack rate

Of the 6,028 contacts traced and entered in the database, 248 became secondary cases, representing an overall secondary clinical attack rate of 4.1% (95% CI: 3.6–4.6). The secondary clinical attack rate was lower in second generation or later contacts (2.3%, 20/861) (95% CI: 1.4–3.6) than in first generation contacts (4.5%, 234/5,175) (95% CI: 4.0–5.1).

**Figure 3:**
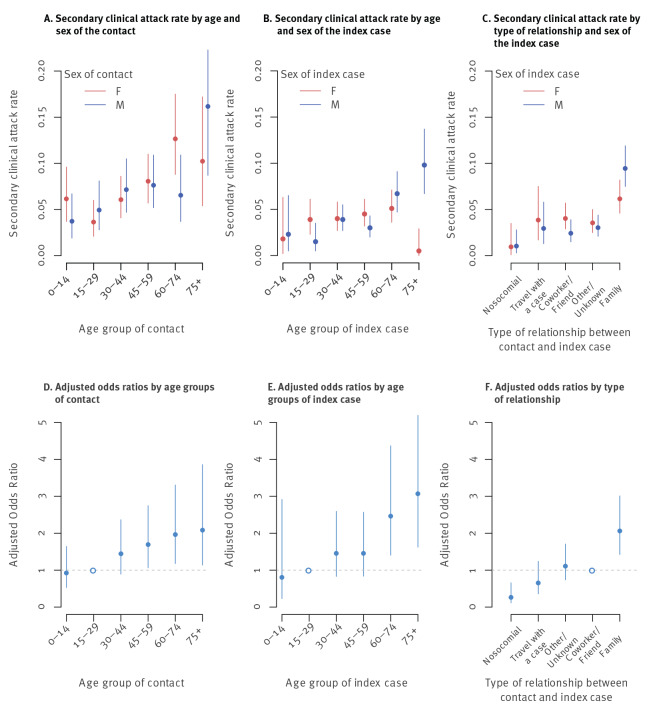
Secondary clinical attack rate and factors associated with the risk of a contact of a COVID-19 case to become a case. France, 24 January 2020–30 March 2020 (n = 735 index cases; 6,028 contacts; 248 secondary cases)

The secondary clinical attack rate decreased over time, from 6.8% at the beginning of the epidemic to 1.8% in weeks 12 to 13 (Figure 2C), and varied by region, from 0% to 8.7% (Figure 2D). The secondary clinical attack rate increased with the age of the contact irrespective of sex, ranging from 4.7% (95% CI: 3.2–6.7) for contacts aged 0–14 years to 12.2% (95% CI: 8.0–17.7) for contacts who were 75 or older (Figure 3A). The secondary clinical attack rate also increased with the age of the index case, from 2.0% (95% CI: 0.7–4.7) for cases aged 0–14 years to 6.2% (95% CI: 4.3–8.7) for cases 75 and older, although there were few index cases aged 0-14 years (n = 15) (Figure 3B). The secondary clinical attack rate did not vary with the sex of the index case, although it was higher for males 75 or older (9.8%) (95% CI: 6.7–13.7) than for females 75 or older (0.5%) (95% CI: 0.0–2.9). The secondary clinical attack rate was the highest among family members (7.9%) (95% CI: 6.6–9.3), followed by co-workers/friends (3.4%) (95% CI: 2.5–4.4), those travelling with a case (3.4%) (95% CI: 1.9–5.4) and nosocomial contacts (1.1%) (95% CI: 0.4–2.3) (Figure 3C).

COVID-19: coronavirus disease.

Reference group for the adjusted odds ratios in panel D and E: 15–29-years-olds. Reference group for the adjusted odds ratios in panel F: co-worker/friend.

### Factors associated with the risk of a contact becoming a case

In univariable analysis, the sex of the index case and the contact was not associated with the risk of a contact becoming a case. Therefore, only the type of relationship, the age of the contact and the age of the index case were considered for the multivariable model. All three of these risk factors remained significantly associated with the risk of a contact becoming a case in the multivariable analysis. The odds of becoming a case were highest for contacts aged 45–59 years (adjusted odds ratio (AOR): 1.7, 95% CI: 1.1–2.7), 60–74 years (AOR: 2.0, 95% CI: 1.2–3.3), and older than 75 years (AOR: 2.1, 95% CI: 1.1–3.9), compared with the reference group of 15–29-year-olds (Figure 3D). The odds were higher for contacts whose index case was 60–74 years (AOR: 2.5, 95% CI: 1.4–4.4) or older than 75 years (AOR: 3.1, 95% CI: 1.6–5.8), compared with the reference group of 15–29-year-olds (Figure 3E). Contacts of index cases younger than 15 years had a similar risk than contacts of index cases aged 15–29 years (AOR: 0.8, 95% CI: 0.2–2.9). We found no significant interaction between the age of the index case and the age of the contact. Family contacts were at higher risk of becoming cases (AOR: 2.1, 95% CI: 1.4–3.0) and nosocomial contacts were at lower risk (AOR: 0.3, 95% CI: 0.1–0.7), compared with co-workers/friends (Figure 3F).

In the sensitivity analyses accounting for regional and temporal differences in data collection, accounting for the generation of transmission or restricting the data to moderate/high-risk contacts, the estimates were not substantially modified compared with the baseline model (Supplementary Figures S2, S3 and S4). The sensitivity analysis that included contacts with multiple index cases (with random assignment of a single index case) also showed results consistent with the baseline analysis (Supplementary Figure S5).

### Chains of transmission

**Figure 4:**
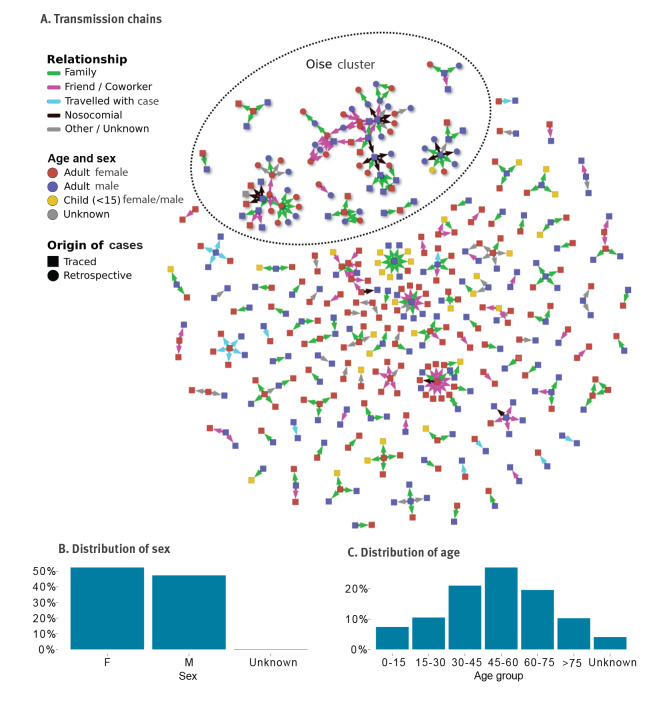
Chains of transmission for COVID-19 cases, distribution of sex and distribution of age. France, 24 January 2020–30 March 2020 (n = 418 cases)

**Figure 5:**
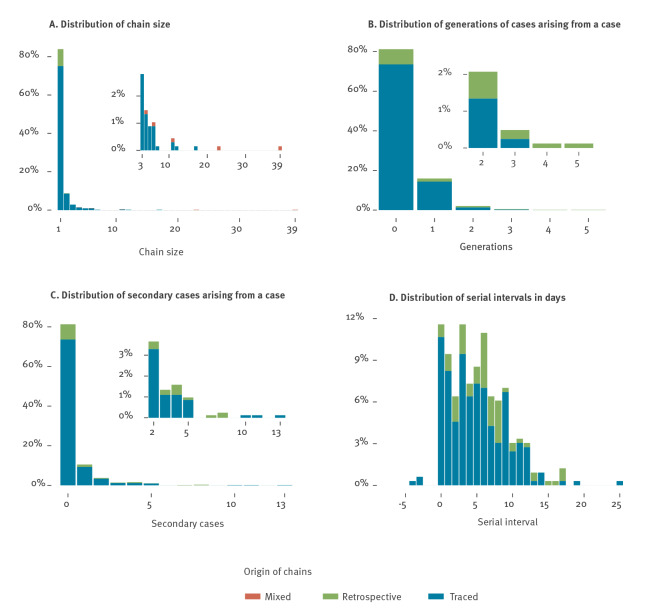
Summary statistics of the transmission chains: chain sizes, generations, secondary cases arising from a COVID-19 case and serial intervals. France, 24 January 2020–30 March 2020 (n = 418 cases)

Overall, 328 connections between cases were identified, representing plausible transmission events between an infector and an infectee (Figure 4). Of these, 259 infector/infectee pairs were identified through contact tracing and 69 were reconstituted retrospectively through epidemiological investigations in Oise. These pairs involved 418 individuals, including 154 infectors. In total, 109 unique transmission chains with at least two cases were identified with a median size of two cases and a mean size of 3.8 cases (Figure 5A). The largest chain included 39 cases and spanned five generations (Figure 5B).

COVID-19: coronavirus disease.

In panel A, only cases involved in a transmission chain of at least two cases are represented.

In panel B, cases with no infected contacts have no generations. Panel C, contact tracing considers all index cases, including those who had no identified transmission chain and are therefore not in the transmission network panel of Figure 4A. For retrospective investigations, this distribution represents all infectors and infectees, including cases with no identified onward transmissions. In panels A, B and C, insets show a portion of the same data after removing the first two bars.

In Oise, the first two cases were identified on 25 February 2020, both with symptom onset on 10 February 2020. Epidemiological investigations showed that these two cases were not the first to occur in the department and were part of a larger cluster, with several large chains of transmission occurring in a military support facility, a secondary school and a high school. Several transmission events were also associated with nosocomial settings such as two hospitals, a clinic and a general practice. In total, nine transmission chains were reconstructed, including the two largest chains – one with 23 cases and one with 39 cases, spanning four and five generations, respectively. Some cases belonging to these chains also gave rise to secondary cases in other departments. A few other transmission events (smaller chains in Figure 4A) were identified in this department during the investigation period but could not be formally linked to the large chains. In total, 97 infector/infectee pairs could be established in this cluster, including 69 through retrospective investigations and 28 through prospective contact tracing.

Among pairs identified through contact tracing, the median age of the infectors was 51 years (interquartile range (IQR) 37–67), 3% (4/127) were children (under 15 years old) and 51% (67/131) were female. The median age of the infectees was 48 years (IQR 30–62), 12% (28/236) were children and 55% (137/247) were female. These characteristics were similar in the retrospective pairs, although the infectees were older than in contact tracing (median age 54 vs 48 years, p = 0.01) (Supplementary Table S3). In the contact tracing pairs, the infectors and infectees were family members in 52% (134/259) of the pairs, coworkers or friends in 26% (67/259) of the pairs, travelling companions in 7% (17/259) of the pairs, associated with nosocomial transmission in 3% (8/259) of the pairs, and “other/unknown” relationships in 12% of the pairs. In the retrospective pairs, the proportion of nosocomial transmission was higher than in contact tracing: 14% (10/69) vs 3% (8/259) (p < 0.001) (Supplementary Table S3).

In the contact tracing data, index cases had 0.34 (95% CI: 0.27–0.42) detected secondary cases on average (Figure 5C). The distribution of secondary cases was highly over-dispersed, with 80% (198/248) of detected secondary cases being caused by 10% (74/735) of cases (negative binomial dispersion parameter (NBDP): 0.17, 95% CI: 0.12–0.22). In retrospective investigations, the mean number of secondary cases was 0.9 (95% CI: 0.53–1.54) and 80% (46/58) of detected secondary cases were caused by 16% (10/64) of cases (NBDP: 0.28; 95%CI 0.09–0.47). Six superspreading events were associated with three infectors generating seven to eight secondary cases (identified through retrospective investigations) and three infectors generating 10–13 secondary cases (identified through contact tracing). These transmission events occurred in the workplace (10 cases), during a dinner between neighbours (six cases), during a family/religious gathering (10 cases) or included mixed types of relationships such as family, nosocomial and co-worker contact (31 cases). Among the 328 reported serial intervals (Figure 5D), 3 (0.9%) were negative and 38 (12%) were null. The serial interval had a mean of 5.1 days (standard deviation 4.1, median 5, IQR: 2–8 days) when calculated on pairs from contact tracing, and a mean of 6.8 days (standard deviation 4.5, median 6, IQR: 3–8) when calculated on pairs from retrospective investigations. Overall, the serial interval decreased over time (Supplementary Figure S6), potentially because of a quicker isolation of cases and/or a right censoring effect (the long serial intervals were not observed at the end of the study period).

## Discussion

Using data from contact tracing and epidemiological investigations conducted during the initial phase of the COVID-19 pandemic in France, we characterised the secondary clinical attack rate and the factors associated with the risk of a contact becoming a case among the 6,082 contacts of the 735 index cases. We also analysed chains of transmission and estimated key parameters of spread among 328 infector/infectee pairs.

Overall, 4.1% of contacts identified and entered into the database became secondary cases. Since only symptomatic contacts were tested and some people infected by SARS-CoV-2 do not develop symptoms [19,20], some asymptomatic infections were probably missed among contacts. As around 20% of SARS-CoV-2 infections are asymptomatic [19,20], we hypothesise that about 60 asymptomatic secondary infections might have been missed among contacts and that about 5% of the contacts (rather than 4.1%) might have been identified as secondary infections if asymptomatic contacts had been tested. In addition to the management of asymptomatic infections, the definition of a contact might vary between studies and therefore impact the estimates of secondary attack rates. Cheng et al. found a lower secondary clinical attack rate of 0.7% [10], but the number of contacts identified per index case was much higher (27 on average, compared with eight in our study) and may have included more low-risk contacts than in our study; in our study, most contacts identified and recorded in the database were moderate/high-risk contacts. Conversely, in studies where 5–10 contacts were identified per index case during the early pandemic, the secondary attack rate (including asymptomatic patients) was 3.7–11.7% [6,7,9,12,21]. More generally, a high variability was observed in the secondary attack rates reported in the literature as the result of different levels of control of the COVID-19 pandemic between countries or different study settings (e.g., contact tracing in the general population vs school settings [22] or early reports of secondary transmission associated with specific events such as meals or holidays [23]).

We found the highest secondary clinical attack rate among family contacts (7.9%, including household and non-household members, which we could not distinguish), highlighting the substantial risks associated with SARS-CoV-2 transmission between close family members [24]. Family contacts might also be easier to identify as they constitute a close and well defined population compared to other types of contacts such as co-workers or friends, who may be more difficult to define. In contact tracing studies where asymptomatic patients were tested, the household secondary attack rate was found to be between 9 and 17% [6,8,9,11,12], but a more recent study found attack rates as high as 53% [25]. As in other studies, the secondary clinical attack rate was lower among nosocomial contacts [7,9,10,12]. We also found a lower secondary clinical attack rate among contacts of the second generation or later, compared with first generation contacts, suggesting the positive impact of isolation measures.

In our study population, we found that the risk of becoming a case was more than twice as high for contacts older than 45 years compared with contacts 15–29 years old. This difference might be because older individuals develop more severe symptoms and therefore are more likely to be detected [26,27]. Since contact tracing is triggered by the detection of a symptomatic case and most infected children appear to be asymptomatic or mildly symptomatic [28], only 15 index cases of 735 were children in our study. Given this important selection bias and the small number of children, our data do not make it possible to robustly compare the infectiousness of children with the infectiousness of adults during the early stages of the pandemic.

Interestingly, the age-specific contact patterns observed in our study before cases are isolated were very consistent with those obtained in a large-scale population survey conducted in France in 2012 [16]. This similarity suggests that the contact tracing data are representative of contact patterns in the general population, and that the Béraud’s contact matrix is appropriate for modelling the early dynamics of a pandemic, before lock-down, as was, for example, done by Salje et al. [26].

We found an average serial interval of 5.1 days in contact tracing pairs, which is consistent with published estimates of 4–6 days obtained in similar contexts of case isolation [6,29-33]. This serial interval must be considered a lower bound of what would happen in a situation without control measures as the isolation of cases has a truncating effect. Such truncation has been demonstrated by Bi et al. in their observation that the serial interval increased with delays in isolating cases, from 3.6 days if the infected individual was isolated less than 3 days after infection to 8 days if the infected individual was isolated on 3 days or later after symptom onset [6]. This finding is consistent with our estimate of 6.8 days for the serial interval in pairs of infectors/infectees, who were identified retrospectively and more likely to have delayed or limited isolation than contact traced pairs.

The mean number of secondary cases identified per index case was 0.3–0.9. These values are lower than the estimates for the reproduction number of SARS-CoV-2 in the absence of interventions, which is around 2.5–3 [26]. This difference can be explained by a combination of factors: the number of secondary cases could be reduced due to contact tracing and isolation measures; asymptomatic infections are not observed in the study settings; and some symptomatic secondary cases might have been missed or not recorded in the database despite contact tracing. Other studies conducted in a similar context obtained similar results, with 0.4–0.7 secondary cases identified per index case [6,8,13]. We estimated the dispersion parameter of the secondary cases’ distribution to be between 0.15 and 0.30, indicating a high degree of superspreading. This result adds to the growing evidence that transmission of SARS-CoV-2 is highly over-dispersed [13,34-36]. This finding has important implications for control efforts – e.g., interventions targeting settings where superspreading events occur could substantially reduce overall transmission.

Our study has several limitations. Data collected during outbreaks are often noisy and incomplete because of the difficult conditions in which they are collected. Case definitions and protocols evolved during the study period to adapt to the changing epidemic situation and new knowledge about the virus and its transmission. More importantly, there are major practical challenges associated with contact tracing, including the difficulty of both identifying all potential contacts of an individual and closely monitoring those contacts during the recommended follow-up period of 14 days, especially with limited human resources. We showed that the proportion of traced cases and the secondary clinical attack rate declined over time and varied across regions. This decline and variation likely reflects variations in data quality and completeness rather than the true evolution of the epidemiological situation due to control measures. Indeed, contact tracing could not be scaled up to meet the exponentially growing burden during the early phase of the COVID-19 pandemic, and investigation teams were quickly overwhelmed with the increasing numbers of new cases. Therefore, the proportion of secondary cases that were missed by the contact tracing was probably larger at the end of the study period than at the beginning, when contacts were easier to trace. Moreover, the work overload was heterogeneous between regions, depending on the local epidemiological situation, and therefore data may vary in quality and consistency. North-eastern France was the most severely affected area (especially Ile-de-France and Grand Est regions) [26]. However, in a sensitivity analysis, our risk factor estimates were robust when accounting for regional and temporal differences in data collection. Another difficulty was collecting data on contacts and cases in healthcare settings. Given that the priority for hospital staff was patient care, it is perfectly understandable that, with the increasing numbers of new patients, only a short amount of time was left for providing epidemiological data and contact tracing. Consequently, a substantial proportion of contacts and secondary cases in hospital settings may have been missed, potentially leading to an under-estimation of the role of the hospital in SARS-CoV-2 transmission. Interestingly, nosocomial transmission was documented in 3% of the infector/infectee pairs identified through contact tracing but was 14% in retrospective pairs in Oise, where thorough investigations probably helped to better document nosocomial transmission. The contribution of healthcare settings to overall transmission seems highly variable across studies [7,9,10]. It was not possible to investigate healthcare workers as a distinct category as they could be classified as co-workers or nosocomial contacts and could not be distinguished (Supplementary Text S2). Finally, the infector/infectee pairs were established by the investigators based on the known relationships between the index and secondary cases and the circumstances of their contact. However, these putative transmission events are not biologically proven, and we cannot exclude that some cases have been exposed to other infected persons. In addition, since not all cases had their contacts traced, the transmission chains that we observed are incomplete and their size underestimated.

In conclusion, this study has contributed to improving our knowledge and understanding of COVID-19 during the early pandemic. Despite the immense efforts necessary to perform contact tracing during outbreaks, collection and analysis of contact tracing data are needed to understand disease transmission and to define effective control strategies.

## Supplementary Data

725000 Supplementary Material
