# Supplementary material for: Early chains of transmission of COVID-19 in France, January to March 2020
Source: Euro Surveill. 2022 Feb 10;27(6):2001953. doi: 10.2807/1560-7917.ES.2022.27.6.2001953 (PMC8832523; doi:10.2807/1560-7917.ES.2022.27.6.2001953)
Supplement: Supplementary Material [file 20-01953_PAIREAU_Supplementary_material.pdf]

Supplementary material

This supplementary material is hosted by *Eurosurveillance* as supporting information alongside the article 'Early chains of transmission of COVID-19 in France, January to March 2020', on behalf of the authors, who remain responsible for the accuracy and appropriateness of the content. The same standards for ethics, copyright, attributions and permissions as for the article apply. Supplements are not edited by *Eurosurveillance* and the journal is not responsible for the maintenance of any links or email addresses provided therein.

Text S1: Retrospective investigations

Cases were interviewed about the contacts they had during the 14 days prior to symptom onset: contacts with sick individuals, contacts with individuals returning from a trip in a risk zone, or participation to any kind of gathering during a prolonged time period. The contact individuals identified through these interviews were then reached to confirm their link with the case, and included as cases in the study if they had symptoms. The pairs were considered as infector/infectee pairs if their characteristics (time between exposure and symptom onset, incubation period, duration and nature of the contact…) were compatible with SARS-CoV-2 transmission based on current knowledge. Data collected through these investigations were entered in the same database as cases and contact-tracing data.

Text S2: Multivariable logistic regression model and sensitivity analyses

We investigated the factors associated with the risk of a contact becoming a case (i.e. developing symptoms and testing positive) using multivariable logistic regression. The model considered associations with age (categorized in 15-year age groups with 15-29 years old as reference), sex and type of relationships. We defined five categories for the type of relationships: (1) family, (2) coworkers (including teachers/students), friends or acquaintance (including neighbours and people regularly sharing leisure, community or religious activities), (3) travel with a case (i.e. the index case and the contact travelled together or shared the same transportation), (4) nosocomial contact (contact in a hospital, a general practice or other healthcare facility), (5) other/unknown. We classified as nosocomial the relationships that were both in family and nosocomial categories, as family the relationships that were both in family and travel categories, and as coworkers/friends the relationships that were both in coworkers/friends and travel categories. There was no specific category for healthcare workers (HCW); they were likely classified as nosocomial contact for HCW-patient relationships and as co-workers for HCW-HCW relationships. Variables with a p-value less than 0.20 in univariable analyses were included in a multivariable model and iteratively removed using backward selection until all variables in the model had p-values less than 0.05. We performed four sensitivity analyses (SA) to assess the robustness of our model. First, we included the region of the index case and the time period as a random effect, in order to assess how our estimates were modified when accounting for regional and temporal differences in contact tracing (SA1). The five time periods considered were the same as in Figure 2: W03-W08, W09, W10, W11 and W12-13 (post-lockdown). Second, we restricted the analysis to moderate/high-risk contacts only (SA2). Third, we adjusted on the generation of transmission (first generation *vs* second generation or later) (SA3). Fourth, fifty-three contacts had multiple index cases (52 had two index cases and one contact had three index cases). These contacts were

removed from the baseline analysis. We evaluated the sensitivity of risk-factor estimates to inclusion of these contacts, with random assignment of a single index case (SA4).

Text S3: Analysis of infector/infectee pairs

We analysed all infector/infectee pairs, using pairs identified through prospective contact tracing (pairs between an index case and a contact who became a case) and pairs identified through retrospective epidemiological investigations in Oise. We described the transmission network in terms of chain size (number of connected nodes) and number of generations. We computed the mean number of secondary cases generated by each case, either based on contact tracing data (using the number of secondary cases observed among traced contacts of each index case), or retrospective data (where cases terminal to the observed chain of transmission were considered to have zero secondary cases). We also fitted a negative binomial distribution to the mean number of secondary cases and estimated the over-dispersion parameter. To deal with secondary cases that had multiple potential infectors, we used a multiple imputation approach: we performed 100 imputations draws, by randomly assigning an infector to these cases, and calculated the pooled mean and 95% confidence interval using Rubin's rules (1). Finally, the serial interval (time interval between symptom onset in infector and infectee) was assessed using the symptom onset dates of (i) pairs identified through contact tracing, and (ii) pairs identified through retrospective investigations. Secondary cases with multiple potential infectors were removed from the serial interval calculations.

Table S1: Case definition of COVID-19, valid from 17 to 29 January 2020

Classification	Definition
**Possible case**	a) Any patient with clinical signs consistent with severe acute lower respiratory infection requiring admission to hospital with no other etiology that fully explains the clinical presentation AND with a history of travel to or residence in the city of Wuhan, Hubei Province, China, in the 14 days prior to symptom onset.<br>b) Any patient with any acute respiratory illness, whatever the severity, AND with history of at least one of the following exposures in the 14 days prior to illness onset:<br>● close contact with a confirmed case of COVID-2019, while symptomatic;<br>● having shared the same risks of exposure as a confirmed case of COVID-2019 (i.e. same history of travel to or residence in the city of Wuhan, Hubei Province, China);<br>● having worked or attended a health care facility where patients with COVID-2019 have been reported;<br>● having visited or worked in a live animal market in Wuhan, Hubei Province, China.<br>c) Any patient with severe acute respiratory infection for whom an etiology that fully explains the clinical presentation has been initially identified, who develops an unexpected clinical course deterioration AND with a history of travel to or residence in the city of Wuhan, Hubei Province, China, in the 14 days prior to symptom onset.
**Confirmed case**	A possible case with a positive SARS-CoV-2 RT-PCR on respiratory samples, performed by an accredited laboratory.

Table S2: Case definition of COVID-19, valid from 13 to 30 March 2020

Classification	Definition
Possible case	a) Any patient with clinical signs consistent with acute respiratory infection with fever or feeling of fever AND with a history of travel to or residence in a risk area, in the 14 days prior to symptom onset.<br>b) Any patient with:<br>● a pneumonia for which another etiology has been excluded based on clinical, radiological and/or virological criteria, and requiring admission to hospital, OR<br>● signs of acute respiratory distress up to ARDS (acute respiratory distress syndrome) in a possibly viral context and without any other obvious etiology.
Probable case	Any patient with clinical signs consistent with acute respiratory infection in the 14 days following close contact with a confirmed case.
Confirmed case	Any patient, symptomatic or not, with a sample confirming SARS-CoV-2 infection.

Table S3: Comparison of case characteristics and type of relationships between contact tracing and retrospective investigations.

	Contact tracing		Retrospective investigations	
	**Infector**	**Infectee**	**Infector**	**Infectee**
**Case characteristics**				
Median age, years (IQR)	51 (37-67)	48* (30-62)	47 (34-60)	54* (46-68)
Proportion of children < 15 years old, % (n/N)	3 (4/127)	12 (28/236)	0 (0/24)	2 (1/57)
Proportion of female, % (n/N)	51 (67/131)	55 (137/247)	52 (13/25)	41 (24/58)
**Type of relationships**				
Family, % (n/N)	52 (134/259)		42 (29/69)	
Coworker/Friend, % (n/N)	26 (67/259)		33 (23/69)	
Travel with a case, % (n/N)	7 (17/259)		0 (0/69)	
Nosocomial, % (n/N)	3* (8/259)		14* (10/69)	
Other/Unknown, % (n/N)	12 (33/259)		10 (7/69)	

* Differences are statistically significant (p<0.05)

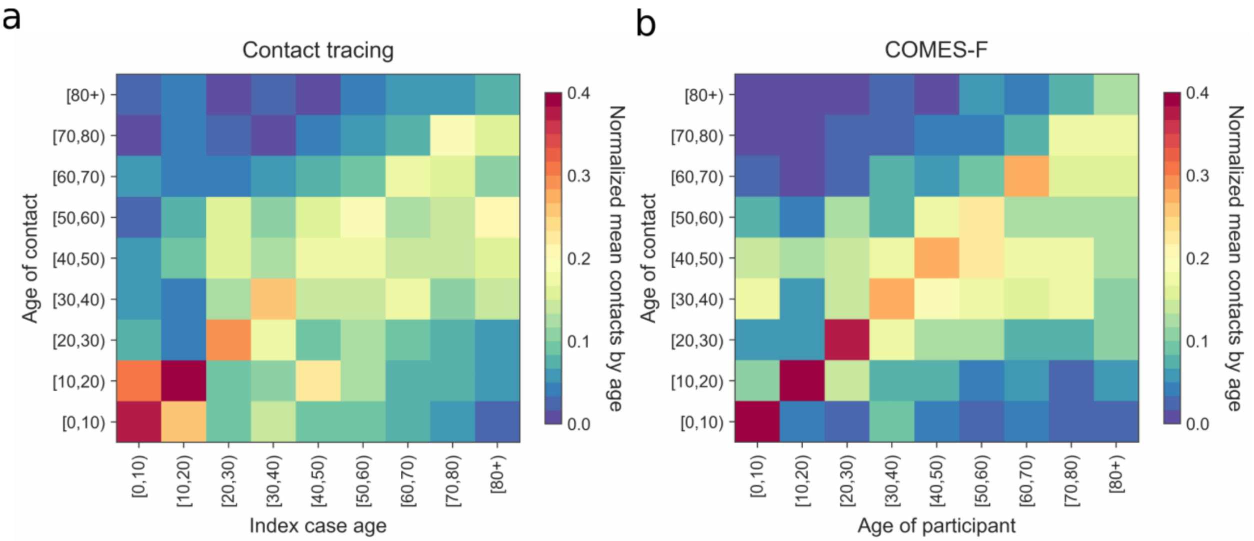

Figure S1: Contact matrices from contact-tracing data and COMES-F study: the entries of each contact matrix correspond to the probabilities for each age group to have a contact with another age group. (a) Contact-tracing data. (b) COMES-F study.

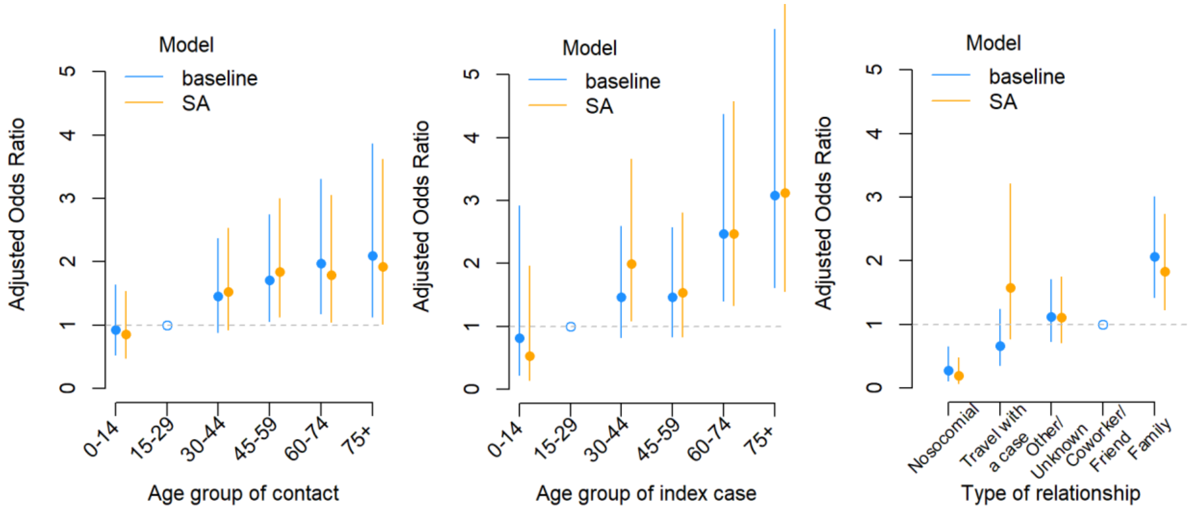

Figure S2: Results of the sensitivity analysis (SA1) including the region of index cases and the time period in the multivariable model. Adjusted odds ratios of the association between contact becoming a case and contact age (reference: 15-29 years old) (A), index case age (reference: 15-29 years old) (B) and type of relationship (reference coworker/friend) (C). When accounting for regional and temporal differences in data collection, the estimates were not substantially modified compared to the baseline model. The two main differences were an increase in the adjusted odds ratio for contacts whose index cases is 30-44 years old, from 1.5 (95%CI 0.8-2.6) in the baseline analysis to 2.0 (95%CI 1.1-3.7) in the sensitivity analysis, and an increase in the adjusted odds ratio for contacts travelling with a case, from 0.7 (95%CI 0.4-1.2) in the baseline analysis to 1.6 (95%CI 0.8-3.2) in the sensitivity analysis, but confidence intervals were overlapping.

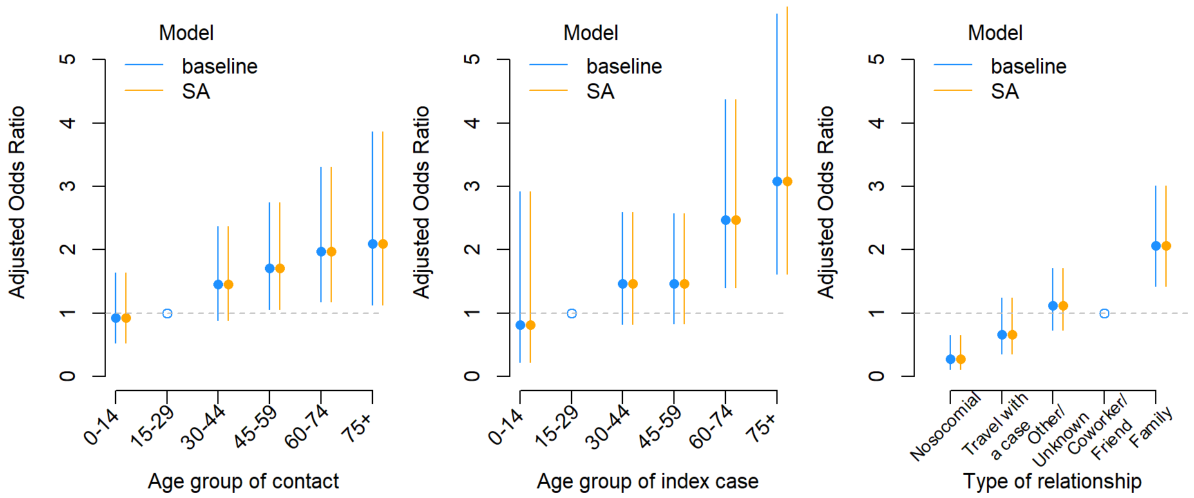

Figure S3: Results of the sensitivity analysis (SA2) restricting the data to moderate/high-risk contacts only. Adjusted odds ratios of the association between contact becoming a case and contact age (reference: 15-29 years old) (A), index case age (reference: 15-29 years old) (B) and type of relationship (reference coworker/friend) (C). Restricting the analysis to moderate/high-risk contacts did not affect the estimates.

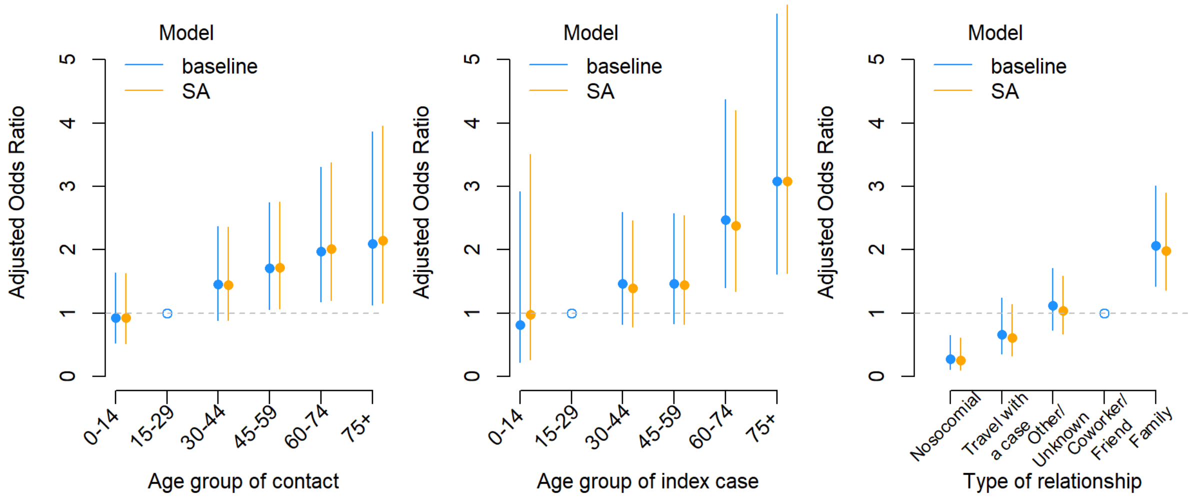

Figure S4: Results of the sensitivity analysis (SA3) adjusting on the generation of transmission (first generation *vs* second generation or later). Adjusted odds ratios of the association between contact becoming a case and contact age (reference: 15-29 years old) (A), index case age (reference: 15-29 years old) (B) and type of relationship (reference coworker/friend) (C). Adjusting on the generation of transmission did not affect the estimates. The odds of becoming a case were lowest for contacts of the second generation or later, compared to the first generation contacts (adjusted odds ratio 0.5 (95%CI 0.3-0.9)).

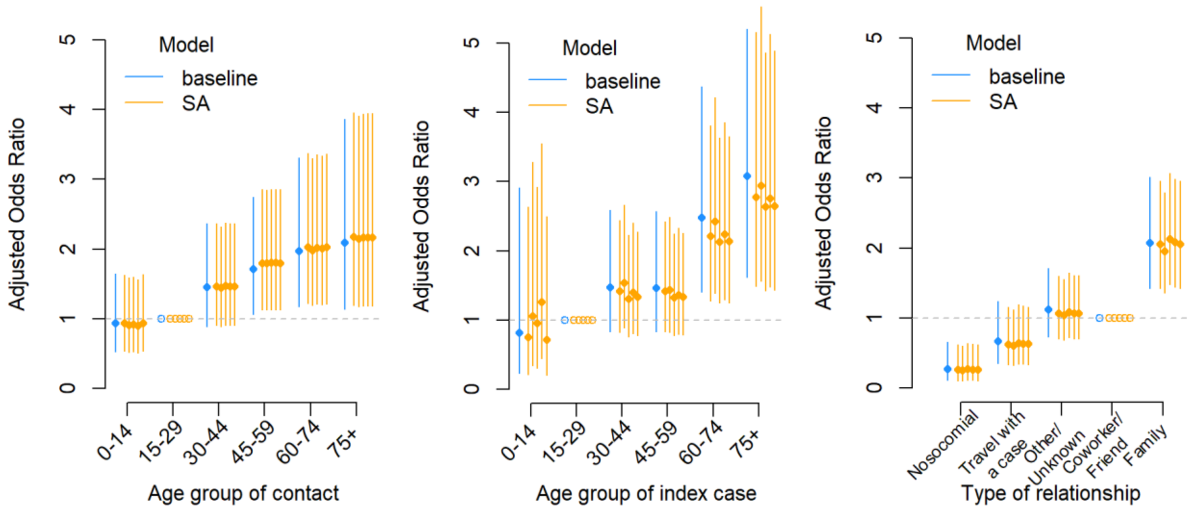

Figure S5: Results of the sensitivity analysis (SA4) on contacts with multiple index cases. Adjusted odds ratios of the association between contact becoming a case and contact age (reference: 15-29 years old) (A), index case age (reference: 15-29 years old) (B) and type of relationship (reference coworker/friend) (C). Fifty-three contacts had multiple index cases (52 had two index cases and one contact had three index cases). We evaluated the sensitivity of risk factor estimates to inclusion of these contacts by random assignment of a single index case. In the three panels, the five orange points correspond to the estimates obtained on five different datasets, in which one single index case randomly drawn among the multiple index cases of a contact was assigned to this contact. In the five scenarios, the estimates were not substantially modified compared to the baseline model.

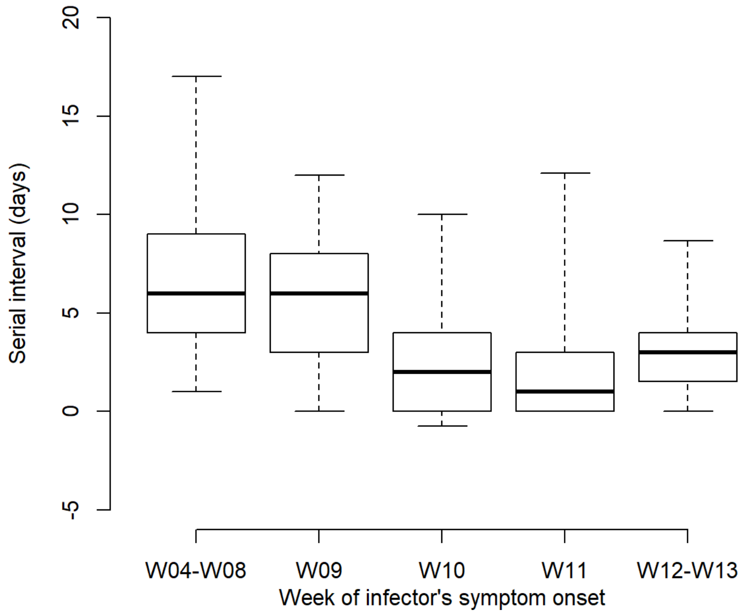

Figure S6: Evolution of the serial interval over time. The horizontal line represents the median, the boxes represent the first and third quartiles, and the whiskers represent the 2.5^{th} and 97.5^{th} percentiles. The national lockdown was implemented on week 12.

References

1. Rubin D. Multiple Imputation for Nonresponse in Surveys. New York: Wiley; 1987.
